# Evaluation of Hepatic Progenitor and Hepatocyte-Like Cell Differentiation Using Machine Learning Analysis-Assisted Surface-Enhanced Raman Spectroscopy

**DOI:** 10.34133/bmr.0190

**Published:** 2025-05-07

**Authors:** Sanghwa Lee, Eunyoung Tak, Jiwan Choi, Seoon Kang, Kwanhee Lee, Jung-Man Namgoong, Jun Ki Kim

**Affiliations:** ^1^ Biomedical Engineering Research Center, Asan Medical Center, Seoul 05505, Republic of Korea.; ^2^ Department of Convergence Medicine, Brain Korea 21 Project, University of Ulsan College of Medicine, Seoul 05505, Republic of Korea.; ^3^ Department of Surgery, Asan Medical Center, University of Ulsan College of Medicine, Seoul 05505, Republic of Korea.

## Abstract

Technology has been developed to monitor the differentiation process of human mesenchymal stem cells (hMSCs) into hepatocyte-like cells (HLCs) and hepatic progenitor cells (HPCs). These cell lineages, differentiated from MSCs, are ethically unproblematic and are gaining attention as promising cell-based therapies for treating various liver injuries. High-sensitivity, label-free, real-time monitoring technologies integrated with artificial intelligence have been used to evaluate and optimize cell differentiation for enhancing the efficiency of cell therapy delivery. Using an Au-ZnO nanorod array-based surface-enhanced Raman scattering (SERS) sensing chip, cell differentiation from hMSCs to HPCs and HLCs was nondestructively monitored through spectral analysis of cell secretions. Principal component extraction was employed to reduce variables, followed by discriminant analysis (DA). The application of principal component–linear discriminant analysis (PC-LDA), an artificial intelligence algorithm, to spectral data enabled clear grouping of hMSCs, HPCs, and HLCs, with monitoring accuracies of 96.3%, 98.8%, and 98.8%, respectively. Spectral changes observed during the differentiation from hMSCs to HPCs and from HPCs to HLCs over several days demonstrated the effectiveness of SERS combined with machine learning algorithm analysis for differentiation monitoring. This approach enabled real-time, nondestructive observation of cell differentiation with minimal sample labeling and preprocessing, making it useful for sensing differentiation validation and stability. The machine learning- and nanostructure-based SERS evaluation system was applied to the differentiation of ethically sourced MSCs and demonstrated substantial potential for clinical applicability through the use of patient-derived samples.

## Introduction

There has been growing interest in using stem cells to treat liver diseases such as liver fibrosis, cirrhosis, and hepatocellular carcinoma, highlighting their great potential [1]. Stem cells have demonstrated significant efficacy in reducing inflammation, fibrosis, and apoptosis; enhancing hepatocyte proliferative activity; and repairing damaged liver tissue [2,3]. The application of mesenchymal stem cells (MSCs) is particularly promising due to the absence of ethical concerns. MSCs are pluripotent and can differentiate into various cell types, including hepatocytes, chondrocytes, osteocytes, and adipocytes, facilitating the replacement and repair of dead cells in damaged organs. Furthermore, MSCs play a crucial role in signal transduction and immune regulation by secreting growth factors such as vascular endothelial growth factor and keratinocyte growth factor, as well as cytokines.

Hepatic progenitor cells (HPCs), which are bipotent cells, can differentiate into hepatocytes and cholangiocytes within the liver [4]. They contribute to the recovery of liver function in hepatic diseases by replenishing the depleted hepatocyte population and differentiating into functional hepatocytes [5,6]. Consequently, HPCs are often regarded as promising candidates for liver disease treatment due to their ability to regenerate both hepatocytes and bile duct cells, depending on the type of liver damage [7]. Maintaining the bipotent state of HPCs serves as a valuable indicator for their identification and determines their optimal therapeutic application based on the injury type.

Liver transplantation, conducted based on severity due to the shortage of suitable donors, requires patients to use lifelong immunosuppressive medications [8]. Hepatocyte-like cells (HLCs) derived from stem cells resemble hepatocytes in their morphology and express liver-specific genes, offering an alternative to liver transplantation. These cells perform essential liver functions, such as glycogen storage and albumin synthesis [9]. However, similar to liver transplantation, there is a scarcity of suitable donors for liver cell isolation, and the quality of isolated hepatocytes varies. Additionally, cryopreservation, the only method for long-term storage of hepatocytes, inevitably leads to changes in cell structure and function upon thawing [10,11]. Therefore, ensuring a consistent supply of high-quality hepatocytes remains a significant challenge for cell therapy in liver diseases.

Accurately characterizing cells is crucial for selecting the most effective treatment cells based on the disease type and severity. Limitations in stem cell use include variability in therapeutic efficacy and potential side effects due to the presence of undifferentiated or other cell types during differentiation [12]. Hence, thorough monitoring of stem cell differentiation is essential to ensure safety and optimize therapeutic strategies.

Common monitoring techniques, such as reverse transcription quantitative polymerase chain reaction (RT-qPCR), flow cytometry, Western blotting, and immunostaining, are frequently employed to assess the expression of specific cell markers. However, these techniques necessitate cell destruction, rendering the cells unusable for therapeutic purposes and limiting validation to a restricted population when cells are produced in large quantities [13,14].

Raman spectroscopy, an analytical method based on inelastic photon scattering due to molecular vibrations, holds promise for monitoring stem cell differentiation. This technique generates unique spectra by detecting energy dispersion from laser-irradiated samples, reflecting molecular composition and structure. The multiple peaks in the spectra provide information on various molecules. Additionally, recent advancements in nanostructured metal technology have significantly enhanced Raman signals. Specifically, surface-enhanced Raman spectroscopy (SERS) has dramatically improved signal sensitivity by amplifying signal intensity through the interaction between localized surface plasmon resonance on the metal surface and molecular vibrations of the sample. SERS thus allows for the acquisition of Raman spectra at lower analyte concentrations.

Our previous studies on kidney diseases demonstrated that Au-ZnO nanoparticle-based SERS is reliable and highly sensitive for biomolecule detection [15,16]. Furthermore, SERS is effective for the early detection and diagnosis of various cancers and atherosclerosis through blood or urine tests [17,18], and its efficacy has been demonstrated in diagnosing rejection-related diseases in clinical samples [19]. This diagnostic method utilizes the characteristics of Au-ZnO nanomaterials, where nanometer biomarkers are filtered via the nanoporous structure of the sensing chip, achieving high-sensitivity detection through the localized surface plasmon resonance phenomenon acting on trapped molecules. When used for diagnosis based on nanometer markers, these biomarkers are widely distributed in a liquid sample, improving the probability of detection even in a single small drop. Various biomarkers exist in blood depending on size, including circulating cells (tens of micrometers), red blood cells (8 μm), bacteria (1 μm), exosomes (less than a few hundred nanometers), and proteins. By targeting nanometer markers, noise signals from nanofiltering can be substantially suppressed.

Various cell metabolites, including extracellular vesicles, exosomes, nucleic acids, and cytokines, are released during cell culture and differentiation and can serve as biomarkers for in vitro evaluation. Utilizing nanobiomarkers for in vitro cell evaluation and monitoring may facilitate highly sensitive analyses with minimal sample volumes. Particularly, when cell function and differentiation can be evaluated through secreted biomarkers or proteins, nondestructive cell analysis becomes feasible, allowing follow-up monitoring and the use of cells as therapeutic agents.

Advances in artificial intelligence (AI) technology and algorithm development have led to its widespread application across the medical industry, including in medical image processing, risk analysis, and new drug development [20–22]. However, most AI diagnostic methods are based on medical images, leaving other medical data largely unexplored. Therefore, the application of AI technology must be expanded. Machine learning technology can be used to improve diagnostic accuracy, develop sensitive detection techniques, and provide evidence for analyzing and automating multidimensional variables. These features make Raman spectroscopy particularly well suited for AI application. However, because Raman spectroscopy data differ from traditional image-derived data, customized research on Raman spectroscopy technology is essential.

**Fig. 1.**
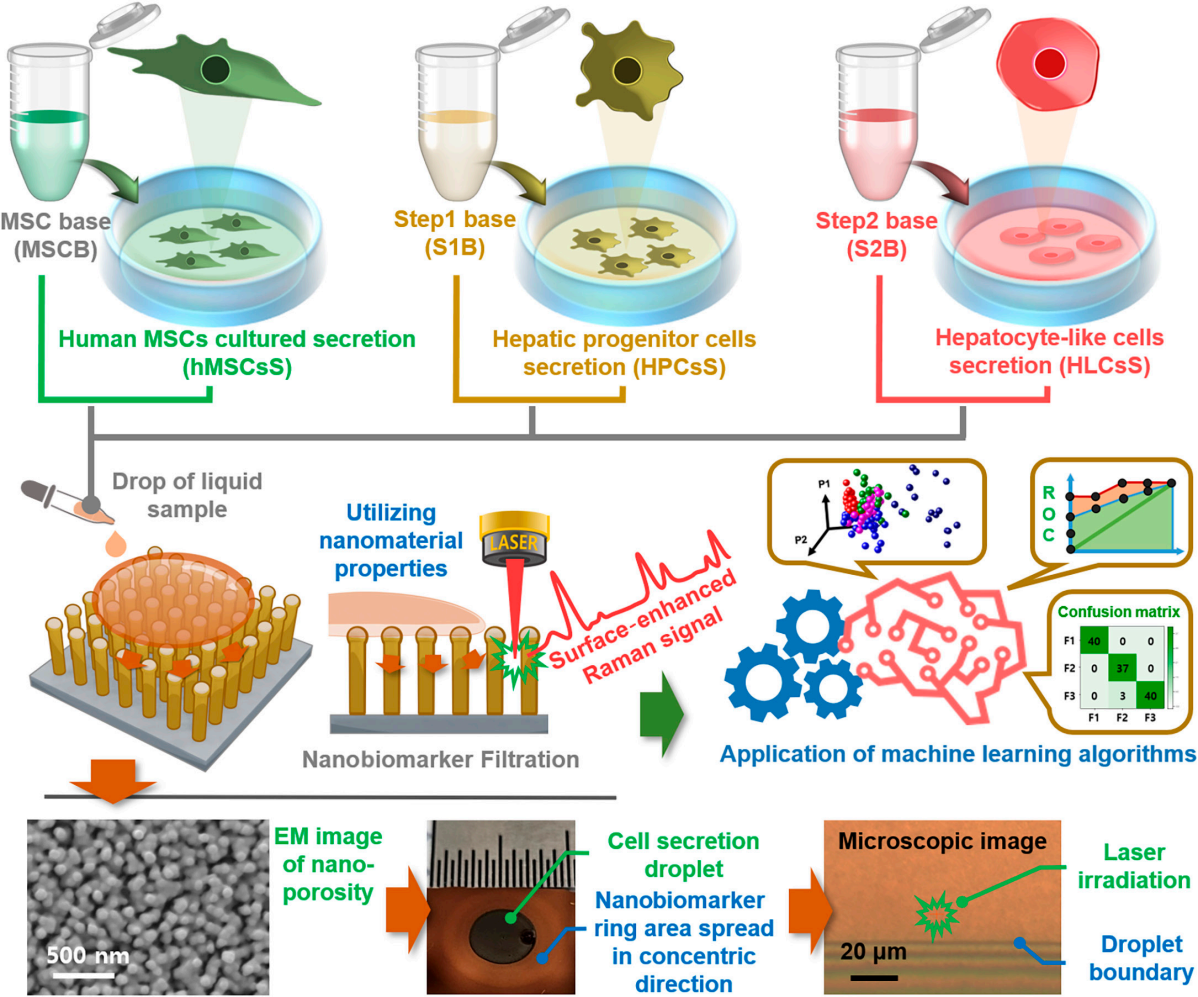
Schematic diagram of the experiment illustrating the identification of nanobiomarkers in the secretion of human mesenchymal stem cells (hMSCs), hepatic progenitor cells (HPCs), and hepatocyte-like cells (HLCs) utilizing surface-enhanced Raman spectroscopy (SERS) integrated with machine learning algorithms for translational research.

In this study, we established culture and differentiation conditions for human MSCs (hMSCs), HPCs, and HLCs for liver disease therapeutics. Validation was performed using conventional biomarker detection methods. Nanobiomarkers were filtered from in vitro samples of culture media and cell secretions, and SERS signals for molecules trapped in the nanoporous structure were measured. The effectiveness of spectral monitoring technology was examined using a machine learning algorithm applied to the obtained Raman spectra, as shown in Fig. 1.

## Materials and Methods

### Conditions of hMSC culture and differentiation into HLCs and HPCs

hMSCs were isolated as described in our previous study [23]. Cells were seeded onto 0.1% gelatin-coated culture plates and cultured in Dulbecco’s modified Eagle’s medium/F12 (Gibco, NY, USA) supplemented with 10% fetal bovine serum (Gibco), 10 ng/ml fibroblast growth factor 2 (PeproTech, Rocky Hill, NJ, USA), 1% nonessential amino acids (Gibco), 1% antibiotic–antimycotic (Gibco), and 0.1 mM 2-mercaptoethanol (Sigma-Aldrich, St. Louis, MO, USA).

The hepatocyte differentiation process was carried out in 2 stages as described previously [24]. Briefly, the stem cells were placed on a 6-well plate coated with 0.1% gelatin. After 2 d, the culture medium was replaced with Step-1 medium, which consisted of Iscove’s modified Dulbecco’s medium (Gibco) supplemented with 0.1% polyvinyl alcohol (Sigma-Aldrich), 10 mM nicotinamide (Sigma-Aldrich), 20 ng/ml human hepatocyte growth factor (PeproTech), 10 ng/ml fibroblast growth factor 2, 2 μM 5-azacytidine (Sigma-Aldrich), 0.1 μM dexamethasone (Sigma-Aldrich), 1% insulin–transferrin–selenium (Gibco), 3 μM CHIR99021, 20 ng/ml human epidermal growth factor (PeproTech), and 10 μM Fasudil (AdooQ Bioscience, Irvine, CA, USA). After 7 d, for hepatic maturation, the Step-1 medium was replaced with the Step-2 medium, which consisted of Iscove’s modified Dulbecco’s medium supplemented with 1 μM dexamethasone, 1% insulin–transferrin–selenium, 20 ng/ml Oncostatin M (PeproTech), 20 ng/ml human hepatocyte growth factor, and 10 μM Fasudil for 14 d.

### Total RNA extraction, RT-qPCR, and detection of secreted human albumin

Total RNA was extracted from all samples using a Qiagen RNeasy Mini Kit (Qiagen, Hilden, Germany) following the manufacturer’s instructions. Approximately 1 × 10^6^ cells were used, and lysis solution (QIAzol, Qiagen) was applied to extract mRNA from the samples. RNA concentration and purity were quantified spectrophotometrically using a NanoDrop (ND-2000, Thermo Fisher Scientific, Waltham, MA, USA).

cDNA synthesis was performed using the ReverTra Ace qPCR RT Master Mix (Toyobo, Osaka, Japan), and RT-qPCR was conducted with 5× HOT FIREPol EvaGreen qPCR Supermix (Solis BioDyne, Tartu, Estonia) using a CFX Connect Real-Time PCR Detection System (Bio-Rad Laboratories, Hercules, CA, USA). Relative expression values were calculated and normalized to glyceraldehyde-3-phosphate dehydrogenase (GAPDH) expression using the ΔΔ*C*_t_ method. Primer sequences are listed in Table 1**.**

**Table 1.** Primer sequence list for real-time PCR detection for each gene

Gene name	Sequences	
	Forward	Reverse
*ALBUMIN*	CACAGAATCCTTGGRGAACAGG	ATGGAAGGTGAATGTTTCAGCA
*CYP3A4*	TTTTGTCCTACCATAAGGGCTTT	CACAGGCTGTTGACCATCAT
*HNF4A*	CAGGCTCAAGAAATGCTTCC	GGCTGCTGTCCTCATAGCTT
*HNF1A*	TGGGTCCTACGTTCACCAAC	TCTGCACAGGTGGCATGAG

The presence of human albumin was determined using an Albumin Human ELISA Kit (Thermo Fisher Scientific) following the manufacturer’s recommendations. Albumin secretion was normalized to the culture days and total cell numbers.

### Synthesis of ZnO nanostructure-based and Au-coated SERS sensing chip

To isolate nanosized biomarkers from the cell culture medium and amplify Raman signals, a SERS chip with a nanoporous structure was fabricated. The SERS chip consisted of ZnO nanorods as the base structure, onto which gold was coated. A ZnO thin film acting as a seed layer was deposited on a silicon wafer to a thickness of 30 to 40 nm. ZnO nanorods, with lengths ranging from 400 to 600 nm and diameters of approximately 50 nm, were grown on this substrate using a hydrothermal method. The ZnO-seeded silicon substrate was immersed in a solution of 25 mM zinc nitrate hexahydrate (Sigma-Aldrich) and 25 mM ammonium hydroxide (Sigma-Aldrich) in 50 ml of deionized water at 90 °C for 50 min. The substrate with the grown ZnO nanorods was coated with a 200-nm gold layer in a thermal evaporator (Alpha Plus, Gyeongbuk, South Korea), as monitored by a thickness gauge. The morphology and nanogap dimensions of the fabricated Au-ZnO nanorods were examined using field-emission scanning electron microscopy (S-4700, Hitachi, Tokyo, Japan) at an acceleration voltage of 10 keV.

### Raman spectrum acquisition and postprocessing of signals

A 5-μl aliquot of cell culture secretion was deposited onto the prepared SERS chip, which was then left in a clean hood for approximately 30 min to allow the biomarkers to diffuse into the nanogaps. The SERS chip with the diffused sample was loaded into a microscope (IX-73, Olympus, Tokyo, Japan) equipped with a Raman spectrometer (FEX-INV, WEVE, Seoul, South Korea), and a 785-nm laser was used to acquire Raman spectra. The Raman signal was calibrated using a 512 cm^−1^ peak from a Si crystal standard sample. For each cell sample group, 20 spectra were obtained from a single drop, covering a spectral range of 350 to 2,400 cm^−1^, with data acquired in 946 steps. Background noise from the raw spectra was removed using fifth-order polynomial fitting, and the signal was smoothed using the Savitzky–Golay method. The average spectrum and standard deviation for each sample group were plotted and compared.

### Statistical analysis of signals and evaluation of classification using machine learning algorithms

Principal components analysis (PCA) was applied to reduce the dimensionality of the Raman spectral data, condensing the 946-step spectral data into 150 components ordered by the highest variability. Based on the validation results of the hMSC, HPC, and HLC groups, true labeling was applied, and predictions were made from the Raman signal data patterns to determine the accuracy of cell culture and differentiation. Linear discriminant analysis (LDA) was performed using the principal components as variables, and the data distribution in the LDA space and confusion matrix was plotted and presented as analysis results. Additionally, receiver operating characteristic (ROC) curves were drawn based on sensitivity and specificity, with accuracy measured by the area under the ROC curve (AUC). PCA and DA were conducted using XLSTAT 2022 software (Paris, France), and all graphs were plotted using Origin 2018 software (OriginLab, Northampton, MA, USA).

## Results

### Description and validation of hMSCs, HPCs, and HLCs by group

**Fig. 2.**
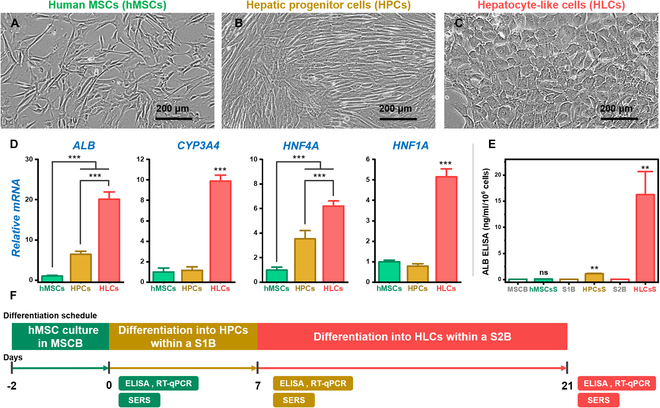
Validation of hepatic differentiation from hMSCs to HLCs. Cell morphology during each (A) hMSC, (B) HPC, and (C) HLC differentiation process. (D) RT-qPCR analysis of hepatocyte-related genes in each cell group. (E) Detection of human albumin secretion in cells and basal medium for each differentiation step using enzyme-linked immunosorbent assay. *P < 0.05; **P < 0.01; ***P < 0.001. (F) Cell differentiation schedule and evaluation timing.

Before monitoring hepatic differentiation using the Raman signal, we induced hMSCs to form HLCs and validated this differentiation using molecular experimental methods such as RT-qPCR and human albumin enzyme-linked immunosorbent assay (ELISA). The shape of differentiated cells changed from fibroblast-like to ovoid, and the cells exhibited a hepatocyte-like morphology on differentiation day 21 (Fig. 2A to C). In addition to observing changes in morphological characteristics, we evaluated differentiation efficiency by detecting hepatocyte-related genes, such as *ALB*, P450 enzyme (*CYP3A4*), and hepatocyte nuclear factors (*HNF4A* and *HNF1A*) using RT-qPCR. The mRNA levels of all hepatocyte-related genes were significantly up-regulated in HLCs on differentiation day 21 (*P* < 0.001; Fig. 2D). Moreover, albumin secretion levels were significantly increased (*P* < 0.001; Fig. 2E). These results validated that the hMSCs had differentiated into HLCs.

As described above, molecular analyses, such as RT-qPCR and ELISA, have been most commonly used to evaluate the efficiency of stem cell differentiation [25]. However, existing molecular analyses require various experimental settings, and real-time and follow-up monitoring are impossible because the differentiated cells are consumed during the analysis. Moreover, since it takes from 4 h (ELISA) to over 2 d (P450 enzyme activity test) to verify differentiation efficiency, molecular analyses have the disadvantage of not allowing a quick response to incorrect differentiation. In particular, the PCR measurement process involves sampling and lysing the sample cells to extract mRNA and obtain genetic information. However, the cells consumed during the analysis cannot be used therapeutically, and PCR alone captures only genetic information, making it difficult to obtain insights into cellular metabolic activities and functions such as detoxification. Therefore, functional evaluation of liver-like cells is performed by detecting albumin, urea, and P450 through concurrent measurement of cell secretions using ELISA. In contrast, liver progenitor cells (HPCs) lack inherent functions and known hepatic roles, and to date, no data on vital activities or specific functions have been accumulated.

Although empirical information regarding the presence of differentiated cells can be provided by monitoring HPCs and hepatic-like cells (HLCs) using liver-related genetic markers (Fig. 2D), there remains an absence of data on cell utility and specific secretions. By accumulating information on secretions in addition to genetic data, it is possible to build empirical data that allow for multifaceted evaluation of cells, as well as leveraging the rapid monitoring capability of the SERS technique. Therefore, to overcome the limitations of molecular analysis methods, SERS technology was employed to evaluate stem cell differentiation.

### Obtaining SERS signals and peak assignments according to individual cell culture conditions

**Fig. 3.**
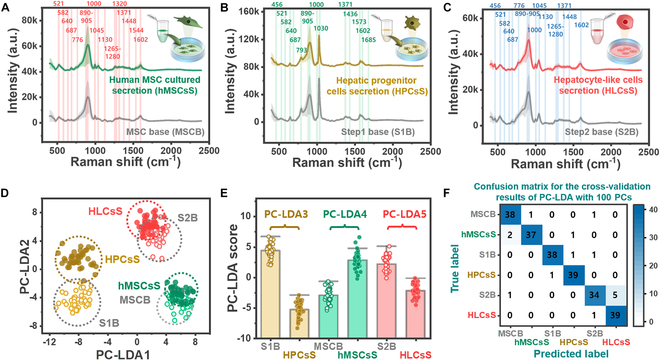
Surface-enhanced Raman spectra of the secretion, with basal medium as the control group, for (A) cultured hMSCs, (B) differentiated HPCs, and (C) HLCs. The average spectrum is plotted as a bold line, with the standard deviation represented as a shaded area of the same color. Main peaks are indicated with semi-transparent colored bars along with their peak values, and peak assignments are shown in Table 2. (D) Data distribution of the overall SERS signals for each differentiation stage in the principal component–linear discriminant analysis (PC-LDA) 1,2 space. (E) Cell sample groups well discriminated using PC-LDA 3, 4, and 5, respectively. (F) Confusion matrix for the cross-validation results of SERS signals classified using the PC-LDA machine learning algorithm.

The approach for detecting nanobiomarkers in liquid samples, as shown in Fig. 1, involves placing a one-drop sample on the prepared nanoporous SERS chip, which is then used to obtain Raman spectral signals. The SERS sensing chip is composed of vertically or slightly tilted arrays of Au-ZnO nanorods on a Si wafer, and the liquid sample is placed as a 5-μl droplet on a chip surface area larger than 1 × 1 cm. The droplet on the sensing chip surface maintains its form because of surface tension, and nanobiomarkers within the liquid sample diffuse concentrically between the nanostructures’ gaps (bottom photo in Fig. 1). When the laser is irradiated onto the area where nanobiomarkers have been filtered and diffused, the Raman scattered light shows enhanced signals only within the tens of nanometer gaps. This signal corresponds to secretions produced during the cell culture process, and the Raman signals are not affected even if large substances, such as cells and vesicles, are introduced during the measurement process. The surface-enhanced Raman signals of secretions derived from the basal medium and cell cultures of hMSCs, HPCs, and HLCs according to each hepatic lineage are plotted in Fig. 3A to C. In these plots, bold lines represent the average spectra obtained by averaging 40 spectra, with regions within the standard deviation shaded in light translucent color. The peaks originating from biomarkers are marked with vertical bars in each spectrum plot, and the Raman shift values are labeled for those peaks. The peak assignments, indicating the chemical information of biomaterials from which these signals are derived, are summarized in Table 2 [26–40]. In Table 2, the main peaks are consolidated to allow for the identification and verification of overlap across different cell types. hMSCs uniquely exhibit peaks corresponding to the C–N bond (1,130 cm^−1^), CH deformation (1,320 cm^−1^), and amide II (1,544 cm^−1^). Meanwhile, HPCs display peaks specific to proline (793 cm^−1^), phenylalanine (1,030 cm^−1^), and amide I (1,685 cm^−1^). In contrast, no unique peaks are observed for HLCs. Notably, since there are no remarkable peak differences in the spectral patterns before and after differentiation within each group, there is a necessity for pattern recognition and classification through machine learning algorithms.

**Table 2.** Peak assignment of Raman spectra obtained from hepatic lineage cells

Peaks (cm^−1^)	Assignment	Related cell types	Ref.
456	L-Tryptophan	HPCs, HLCs	[26]
521	S–S disulfide stretching in proteins	hMSCs, HPCs, HLCs	[27,28]
582	Succinic acid	hMSCs, HPCs, HLCs	[26]
640	Tyrosine	hMSCs, HPCs, HLCs	[29,30]
687	Succinic acid	hMSCs, HPCs, HLCs	[26]
776	Phosphatidylinositol	hMSCs, HLCs	[28,31]
793	Proline	HPCs	[26]
890–905	C–C skeletal of backbone and protein	hMSCs, HPCs, HLCs	[28,32]
1,000	Symmetric ring breathing of phenylalanine	hMSCs, HLCs	[16,18]
1,030	C–H bending of phenylalanine	HPCs	[16,18]
1,045	Proline	hMSCs, HLCs	[28,33]
1,130	Protein C–N stretch	hMSCs	[28,33]
1,265–1,280	Amide III	hMSCs, HLCs	[18,28]
1,320	CH deformation of proteins	hMSCs	[34,35]
1,371	CH_3_ stretching related to cell membrane lipids	hMSCs, HPCs, HLCs	[28,36]
1,436–1,440	CH_3_ deformation related to cell membrane lipids	hMSCs, HPCs	[28,36]
1,448	CH deformation related to cell membrane lipids	hMSCs, HLCs	[28,36]
1,544	Amide II	hMSCs	[17,37]
1,573	Guanine, adenine, TRP (protein)	hMSCs, HPCs	[38]
1,602	C=C bending of phenylalanine	hMSCs, HPCs, HLCs	[17,18]
1,685	Amide I	HPCs	[39,40]

HPCs, hepatic progenitor cells; HLCs, hepatocyte-like cells; hMSCs, human mesenchymal stem cells; SERS, surface-enhanced Raman spectroscopy; TRP, tryptophan

### Application of machine learning algorithms for evaluating the accuracy of cell culture monitoring

To examine the AI recognition performance of the spectral signals, DA was conducted to differentiate the cases shown in Fig. 3A to C. The spectral range of 350 to 2,400 cm^−1^ was sampled in 946 steps, and reduction to 100 variables was achieved using PCA. For PCA-based dimensionality reduction of the spectra (Fig. 3A to C), no additional preprocessing or adjustment of the spectral range was performed. The 890 to 905 cm^−1^ region of the spectrum showed a significant deviation unrelated to the cell groups, whereas other regions exhibited highly reproducible patterns. The principal component (PC) numbers were sorted in ascending order according to the size of the variance, with earlier-ranking PCs being less causally related to distinguishing each cell group. These reduced variables were used to perform LDA, and the data distribution in the PC-LDA1, 2 plane was confirmed, as shown in Fig. 3D. The data for the basal medium and corresponding culture secretions at each cell stage were closely distributed, allowing for visual comparison of their spectral shapes. As shown in Fig. 3E, PC-LDA3–5 serve as criteria for determining the culture status of HPCs, hMSCs, and HLCs, respectively. Ultimately, the base- and cell-specific grouping of the 6 cases was achieved by reducing the variables to a 5-dimensional space represented by PC-LDA1–5. The confusion matrix derived from the PC-LDA showed a perfect match between the true and predicted labels. In addition, the cross-validation results demonstrated the generalization capability of PC-LDA (Fig. 3F), with a high probability of matching true and predicted labels, achieving an accuracy of 93.75%.

**Fig. 4.**
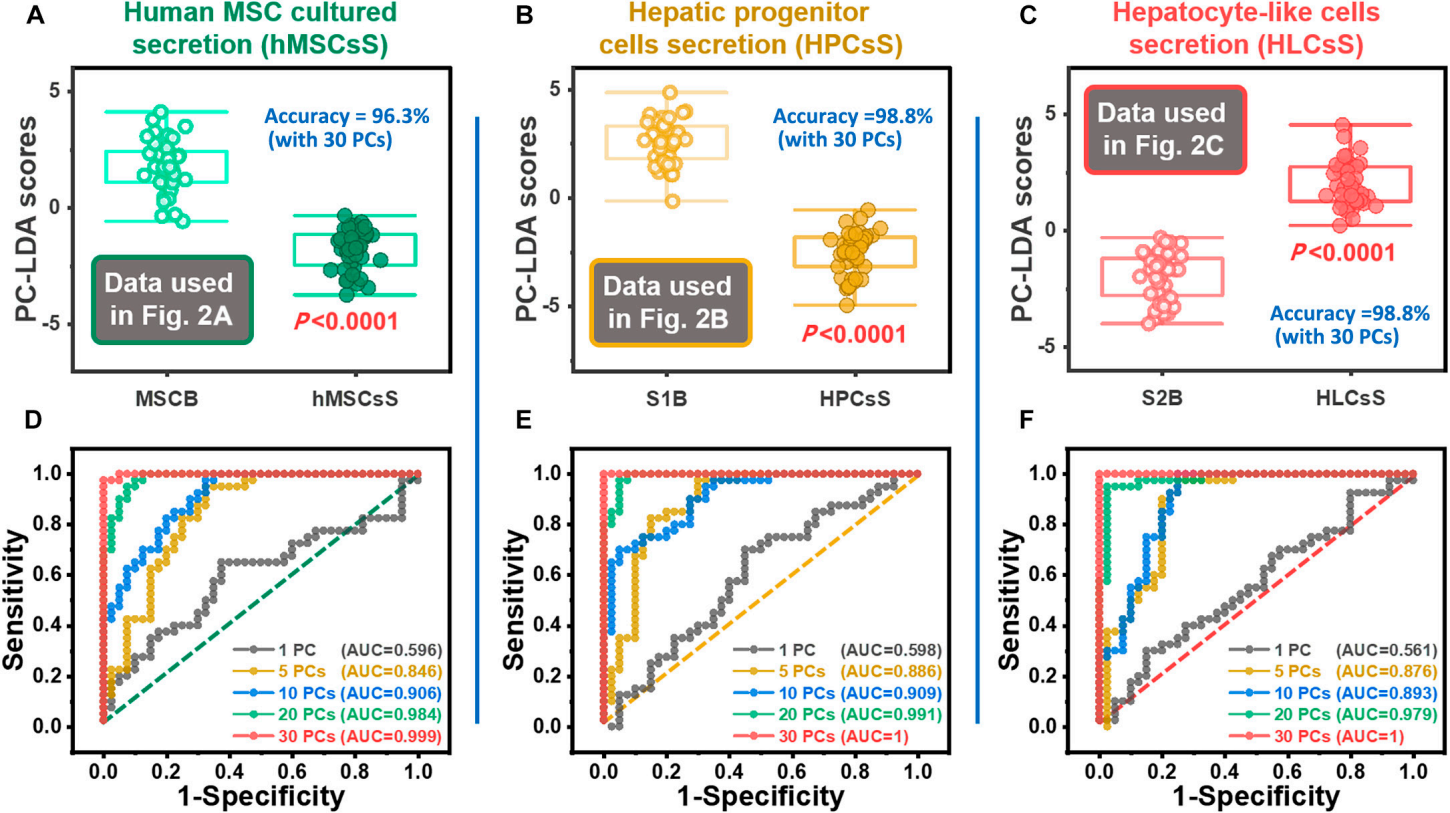
Distribution graphs of Raman data for the secretions and basal medium after cell culture for (A) hMSCs, (B) HPCs, and (C) HLCs separated by PC-LDA. Receiver operating characteristic (ROC) curves and respective area under the curve (AUC) values for (D) human MSC cultured secretion (hMSCsS) versus MSCB, (E) HPCsS versus S1B, and (F) HLCsS versus S2B as a function of the number of PCs used.

To evaluate the prediction accuracy for cell culture based on the basal medium, as illustrated in Fig. 4, accuracy assessment was performed. The data distribution for each case is depicted in Fig. 4A to C, and quantitative accuracy obtained using 30 PCs was 96.3%, 98.8%, and 98.8% for hMSCs, HPCs, and HLCs, respectively. In all cases, the significance level was *P* < 0.0001. Figure 4D to F shows ROC curves plotted while increasing the number of PCs used in DA to 1, 5, 10, 20, and 30. The AUC values for each ROC curve are also presented to show changes in accuracy improvement. The spectra used to derive the mean and standard deviation shown in Fig. 3A were applied to the machine learning algorithm PC-LDA, and the accuracy of the low-dimensional data distribution and discrimination between base media and cell culture secretion is demonstrated in Fig. 4A. Biomolecules contributing to the spectral pattern formation (Fig. 3A) correspond to those of hMSCs listed in the third column of Table 2, which similarly contribute to the accuracy (Fig. 4A). The biomolecules contributing to Fig. 4B and C can be referenced from HPCs and HLCs in Table 2, respectively.

In PCA, standardization of the entire spectrum and extraction of PC vectors were conducted without distinguishing between cell groups. PCs 1 to 3 account for 72.45% of the total variability, indicating a substantial occupation by these top PCs. Furthermore, the PC-LDA conducted on the entire cell group reveals that these PCs 1 to 3 significantly contribute to classification, with *P* values <0.0001, as shown in Fig. 3D to F. In contrast, individual classifications depicted in Fig. 4 show that the sum of *P* values for PCs 1 to 3 is 0.499 for hMSCs, 0.112 for HPCs, and 0.708 for HLCs, indicating lower variability occupation in monitoring culture and differentiation within each cell group. Achieving high accuracy through PC-LDA implies that the machine learning algorithm successfully extracted crucial classification patterns from minor spectral differences.

### Verification of SERS assessment validity for monitoring cellular differentiation processes

**Fig. 5.**
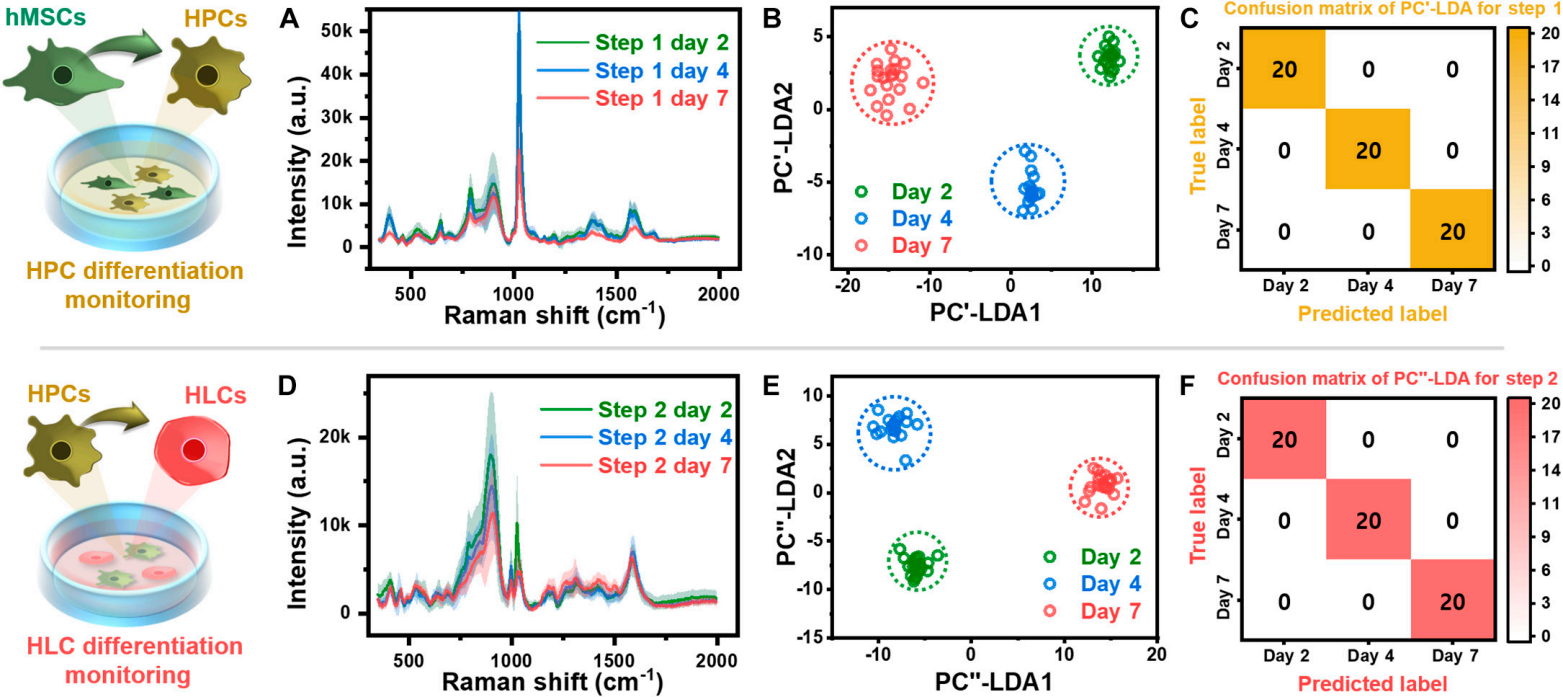
Utilization of SERS signals for monitoring the differentiation process from hMSCs to HPCs and from HPCs to HLCs. Overlapped spectra by day during HPC differentiation: (A) overlapped spectra, (B) data distribution from PC-LDA, and (C) confusion matrix derived from PC-LDA. Overlapped spectra by day during HLC differentiation: (D) overlapped spectra, (E) data distribution from PC-LDA, and (F) confusion matrix derived from PC-LDA.

To monitor the differentiation of hMSCs to HPCs and from HPCs to HLCs, cell secretions were collected on days 2, 4, and 7, and SERS signals were obtained. As shown in Fig. 5A and D, the spectra for each day were overlapped and plotted, with each bold line representing the average spectrum of 20 data points and the standard deviations shaded in the same color. These spectral data are illustrated as distributions in the PC-LDA space in Fig. 5B and E, and confusion matrices shown in Fig. 5C and F. The distributions of the data in Fig. 5B and E revealed very clear grouping for each day, with data within each group closely distributed compared to the distance between group centers. These results indicate that fine stage changes can be monitored, suggesting that accuracy for each time point is high.

The cell secretions used for classification in Fig. 3 are samples collected at the final stages after 2 d of culture and subsequent 7 and 14 d of differentiation in different base environments for hMSCs, HPCs, and HLCs. In contrast, Fig. 5 demonstrates differences in secretions obtained at 2- to 3-day intervals, enabling more detailed temporal monitoring. Specifically, Fig. 5A and D shows that certain spectral regions sequentially decrease over time, indicating that Raman signals from nanobiomarkers serve as credible indices for monitoring stem cell differentiation. Regions showing sequential changes include 365 to 440, 485 to 675, 730 to 970, and 1,515 to 1,645 cm^−1^ for hMSCs differentiating to HPCs, while the 720 to 970 cm^−1^ range is pronounced for HPCs differentiating to HLCs. These findings, highlighted by the sequential changes from SERS chips upon dropping the secretion samples, demonstrate the self-filtering characteristics of nanobiomarkers and validate the feasibility of using SERS for monitoring differentiation.

## Discussion

The liver possesses remarkable regenerative capabilities; however, in cases of chronic disease or severe damage, natural regeneration may be inadequate to fully restore its function. Various liver conditions, such as chronic hepatitis B, chronic hepatitis C, nonalcoholic steatohepatitis, alcoholic liver disease, and cirrhosis, can cause irreversible liver damage, often requiring liver transplantation and potentially progressing to liver cancer. Transplanted livers have a high risk of immune response-related rejection, and ensuring the safety of developing liver therapeutics requires extensive time. Consequently, HLCs and HPCs have emerged as promising cell therapies for treating these diseases. HLCs contribute to liver function recovery by replacing or supporting damaged liver cells, whereas HPCs, capable of differentiating into both hepatocytes and cholangiocytes, promote the regeneration of damaged liver tissue. The homing ability of liver-derived cells makes HPCs and HLCs attractive for cell therapies, allowing them to be targeted to the liver without surgical intervention.

The development of these cell therapies begins with an in vitro stage, where the technology to produce HLCs and HPCs in sufficient quantity and of high quality is crucial. Subsequent steps focus on ensuring transplantation efficacy and safety by enhancing posttransplantation cell engraftment rates and minimizing potential side effects. Ultimately, addressing long-term stability and ethical concerns ensures strict regulatory standards and ethical considerations for the clinical application of cell therapies. In this study, machine learning-based SERS demonstrated high accuracy in monitoring the differentiation of hMSCs into HPCs and HLCs, highlighting its potential in automating and managing the quality control of cell therapy production for clinical applications. The high accuracy observed is attributed to the use of well-established and validated differentiation protocols from previous studies [23]. Our differentiation model showed near-complete differentiation into HLCs by day 14, confirmed by albumin immunofluorescence imaging. Although the Raman patterns obtained in this study were validated with high differentiation rate samples, the method holds major limitations for reverse-tracing differentiation rates. DA results obtained from SERS were based on signals from secretions collected after cell culture, rather than directly from the cells. The base medium on the culture dish was replaced every few days, and the collected secretions were transferred to the SERS detection chip for spectral signal collection during this process. Given that cells concurrently undergo culture and differentiation during these intervals, deriving differentiation rates influenced by accumulated secretions poses significant challenges and limitations in measuring specific parameters. Using systems such as flow cytometry that effectively measure cell differentiation rates in parallel, these limitations can be overcome, enabling evaluation of the significant strengths of AI-based SERS analysis in future studies. However, there are emerging concerns regarding the influence of environmental factors on diagnostic technologies based on Raman spectral patterns, rather than on diagnostic methods that utilize specific binding properties, such as immunoassays. Notably, spectral patterns are formed from substances present in liquid secretions and can be altered by variations in cultivation conditions or subtle environmental changes. For instance, factors such as changes in composition during the cell culture process, metabolic rates associated with different cell types, or environmental conditions such as temperature and pH can affect the composition of biomaterials in liquid secretions. This issue may be addressed by building a dataset through repeated testing under various environmental conditions, enhancing the patterns for monitoring cell differentiation, and diminishing the influence of external factors on spectral forms. Therefore, a remaining challenge is to establish the validity of this diagnostic technology across diverse experimental conditions to improve the generalizability of its performance.

In Fig. 3A to C, the spectra that are difficult to distinguish by visual inspection were classified with the assistance of AI algorithms. Furthermore, since the peaks contributing to the classification were influenced by data from multiple regions, there are limitations in explaining diagnostic evidence based solely on specific peaks assigned as biomarkers. Determining which biomarkers contribute to the culture and differentiation may be feasible through more refined sampling or purified samples; however, deep experimental design is required to secure nanometer-level biomarker candidates and perform precise evaluations. To ensure reliability within the data, cross-validation was conducted as shown in Fig. 3F, wherein a portion of the spectra from the training data was separated into a test set for iterative evaluation. While this approach minimized the errors of overfitting and achieved high classification accuracy, leveraging the data to gain new insights in the context of stem cell differentiation research remains challenging.

Further scientific development on machine learning-based SERS for cell transplantation and therapeutic effects in vivo will be reported in future studies. This study conducted AI analysis on SERS signals derived from metabolic products of differentiating hepatic lineage cells in vitro. Following in vivo transplantation of liver cells, changes in metabolic products are anticipated to be reflected in the blood. In previous studies, kidney injury can be monitored using blood and urine samples [15,16], and kidney transplant rejection can be diagnosed with high accuracy using clinical patient-derived samples containing various factors [19]. The differences in Raman signals from patients, along with various clinical environmental variables, were resolved by applying machine learning algorithms that isolate distinguishing patterns between the disease and control groups. Furthermore, ischemic liver failure biomarkers can also be diagnosed via SERS, and hMSC-derived HPCs exhibit effective homing to the liver [23], fostering optimism for positive in vivo results in the future. However, because enhanced Raman signals are particularly sensitive to chemical environments, patterns that distinguish between disease and control groups can potentially be obscured by noise from other chemical factors. Particularly in clinical environments, various variables can influence Raman signals, so it is crucial to understand these methodological limitations and carefully consider aspects such as spectral data preprocessing and the risks of overfitting. For clinicians, if SERS assessments are performed based on blood obtained from patients with liver disease undergoing pharmacotherapy, spectra derived from the drugs may be interpreted as evidence of the disease. This poses a challenge to generalizing label-free Raman-based diagnostics. The design of an experimental plan that allows for a meticulous observation of clinical signals from spectra obtained in in vitro models and well-controlled preclinical animal models will be key to accelerating the establishment of clinical efficacy. Additionally, since the genes of the MSCs obtained from each clinical patient differ, variations in metabolic products derived from stem cells during the culture and differentiation processes can lead to differences in signals. An evaluation of interpatient variation is also necessary.

To monitor cell differentiation, we integrated a structure that enhanced Raman signals while filtering nanobiomarkers, a Raman spectroscopy technique for acquiring label-free chemical signals, and AI algorithm-based DA. Evidence for differentiation was observed in cell culture secretions, where filtering targeted nanometer markers minimized signal variation in the data. Furthermore, the distinct patterns relevant to differentiation in the spectra derived from nanometer biomarkers were identifiable by machine learning algorithms. This approach was enabled by the convergence of nano, spectroscopic, and AI technologies, leveraging a complementary synergy of their key features. Such a diagnostic platform offers engineering advancements when utilized in clinical settings. The SERS chips used in this study are fabricated on silicon wafers and can reduce measurement costs through unit chip optimization processes such as large-scale production and dicing. Furthermore, the commercialization of a motorized Raman system enables the automation of spectral signal acquisition. As no additional pretreatment other than spot application on the SERS chip is required, it provides a user-friendly system that can be implemented in clinical environments. Given that it is a platform capable of diagnosis through AI analysis, it is expected to be a technology that allows users easy access to the results.

## Conclusion

In summary, we developed a technology for the rapid monitoring of cell culture and differentiation using a single drop of secretion by acquiring SERS signals and applying AI evaluation methods for nanobiomarkers. A sensing chip capable of selectively filtering nanobiomarkers and obtaining SERS signals was fabricated based on an array structure of Au-ZnO nanorods. Additionally, hepatic lineage cells (hMSCs, HPCs, and HLCs) were differentiated and cultured, and their secretions during these processes were acquired as liquid samples for SERS signal collection. Surface-enhanced Raman spectra were obtained in the range of 350 to 2,400 cm^−1^, consisting of 946 steps of biomarker information. The spectra of markers trapped in the nanoporous space were presented with peak assignments, indicating their origin from biological materials. For DA of each cell differentiation stage, the machine learning algorithm PC-LDA was applied for spectrum analysis, confirming data grouping. The accuracy of monitoring the cultures of hMSCs, HPCs, and HLCs was 96.3%, 98.8%, and 98.8%, respectively. During the differentiation of hMSCs to HPCs and HPCs to HLCs, surface-enhanced Raman spectra were obtained on days 2, 4, and 7, and DA was performed using machine learning algorithms. Clear grouping of data by day during the differentiation process was confirmed. These results demonstrate that monitoring of hepatic lineage cell differentiation and culture can be assessed with high accuracy using a single drop of secretion.

## Data Availability

The data are available upon reasonable request.
